# Exposure to famine in early life and chronic kidney diseases in adulthood

**DOI:** 10.1038/s41387-017-0014-9

**Published:** 2018-06-15

**Authors:** Ningjian Wang, Zhiyuan Ning, Fangzhen Xia, Chi Chen, Jing Cheng, Yi Chen, Yingli Lu

**Affiliations:** Institute and Department of Endocrinology and Metabolism, Shanghai Ninth People’s Hospital, Shanghai JiaoTong University School of Medicine, Shanghai, China

## Abstract

**Objective:**

Chronic kidney disease (CKD) is an increasing contributor to the global disease burden. Previous findings indicated that exposure to famine in early life was associated with various metabolic diseases and urinary protein levels. We aimed to assess whether the exposure to China’s Great Famine 1959–1962 during fetal or childhood period was associated with glomerular filtration rate (GFR) and risk of CKD (eGFR<60 mL/min per 1.73 m^2^) in adulthood.

**Materials and methods:**

SPECT-China was a population-based observational study in 2014–2015. Totally, 5124 women were included from SPECT-China study. Based on the birth year, they were divided into fetal-exposed (1959–1962), childhood-exposed (1949–1958), adolescence/young adult-exposed (1921–1948), and non-exposed (1963–1974, reference). The estimated glomerular filtration rate (eGFR) was calculated according to the Chronic Kidney Disease Epidemiology Collaboration equation. CKD was defined as eGFR less than 60 mL/min per 1.73 m^2^.

**Results:**

Compared with the non-exposed, fetal exposure to famine was significantly associated with lower eGFR (B −1.47, 95%CI −2.81, −1.13) and greater risk of having CKD (OR 2.85, 95%CI 1.25, 6.50) in the crude model adjusting age. Further adjustments for demographic variables, body mass index, diabetes, and blood pressure did not qualitatively change the association (eGFR B −1.35, 95%CI −2.67, −0.04; CKD OR 2.42, 95%CI 1.05, 5.58). This association was not found in childhood-exposed and adolescence/young adult-exposed individuals.

**Conclusions:**

Prenatal exposure to famine may have long-term effects on declined GFR and the development of CKD in humans. thus, fetal stage may be an important time window to prevent CKD in later life.

## Introduction

Chronic kidney disease (CKD) is an increasing contributor to the global disease burden. CKD is the underlying cause of death increased by 82% from 1990 to 2010^1^. In addition to these deaths, a declined glomerular filtration rate (GFR) may also increase the risk of death^2^. Whereas, age-standardized death rates of major vascular diseases, chronic obstructive pulmonary disease, and liver cirrhosis showed important decreases (20% or more)^1^. Therefore, CKD may be a leading public health problem in the next several decades. That’s what is happening in China. A national survey in 2010 demonstrated that the overall prevalence of CKD was 10.8%, thus the number of patients with CKD was about 119.5 million in China^3^. It has been known that this phenomenon associates with rapid increase in the prevalence of risk factors such as diabetes, hypertension, and obesity which is common globally^3^, however, nutrition status in early life may be another risk factor.

Developmental programming or the Developmental Origins of Health and Disease postulates that exposure to an adverse intrauterine or early postnatal environment leads to structural and functional changes, and elevates the risk for chronic disease development later in life^4, 5^. China’s Great Famine, lasting from the late 1950s to the early 1960s, was such an adverse environmental event. It affected almost the whole nation and caused 30 million deaths and another 30 million lost births during this period^6, 7^. Compared to the Dutch Famine, it lasted longer, spread in larger areas and affected more people. Thus, it provides a precious chance to study the impact of famine/malnutrition in early life on adult diseases.

There has been increasing evidence that exposure to famine in utero and early postnatal life associates with higher risk of obesity^8^, diabetes^9, 10^, fatty liver^11^, metabolic syndrome^12^, etc. However, there were rather limited studies measuring the association between famine exposure and kidney disease. Only two studies from Dutch Famine and China’s Great Famine found famine exposure during gestation and early postnatal life was associated with higher levels of protein concentration in urine and microalbuminuria^6, 13^. Other studies taking low birth weight (LBW) as the surrogate of prenatal malnutrition found LBW was associated with elevated risk of reduced GFR and albuminuria^14^. However, no study has demonstrated whether famine exposure in early life was associated with declined GFR and higher risk of CKD.

We conducted the Survey on Prevalence in East China for Metabolic Diseases and Risk Factors (SPECT-China) in 2014–2015. We aimed to analyze the relationship among famine exposure in different life stages, estimated GFR (eGFR) and risk of CKD (eGFR < 60 mL/min per 1.73 m^2^) in adulthood.

## Subjects and methods

### Study population

**Fig. 1:**
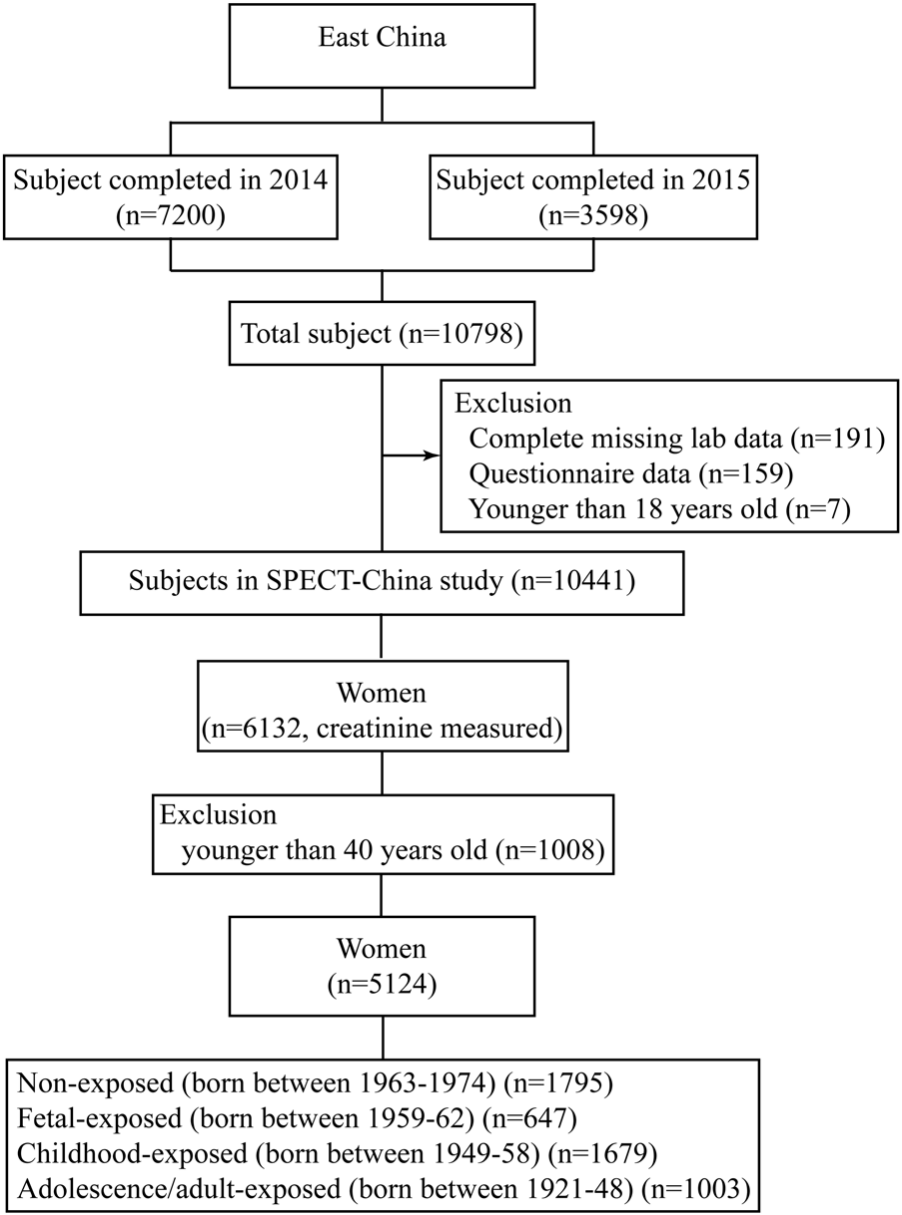
Flowchart of the participants selected from SPECT-China

SPECT-China, which was established from 2014 to 2015, was a cross-sectional survey of the prevalence of metabolic diseases and associated potential risk factors in East China (ChiCTR-ECS-14005052, www.chictr.org.cn). A two-stage stratified cluster sampling method was used. The sampling process was stratified by urbanization (rural/urban area) and economic development status (high/low) (Fig. 1). “Chinese citizens ≥18-years-old who had lived in their current area for ≥ 6 months were selected. We also excluded subjects with severe communication problems, acute illness or who were unwilling to participate. From 2014 January to 2015 December, 10,441 subjects who were 18–93-years-old were recruited in the SPECT-China study from 22 sites in Shanghai, Zhejiang, Jiangsu, Anhui, and Jiangxi Province”^15^. The detailed sampling process has been described in previous studies^9, 10, 12^.

There were 6132 women recruited, where, all of them had creatinine data. The exclusion criteria were the following being younger than 40-years-old (*n* = 1008). Finally, 5124 subjects were included in this famine exposure study (Fig. 1).

The study protocol was approved by the Ethics Committee of Shanghai Ninth People’s Hospital, Shanghai JiaoTong University School of Medicine. All procedures followed were in accordance with the ethical standards of the responsible committee on human experimentation (institutional and national) and with the Helsinki Declaration of 1975, as revised in 2008. Informed consent was obtained from all patients included in the study.

### Measurements

Participants’ demographic characteristics, medical history, and lifestyle risk factors were collected by trained staff. Body weight, height, and blood pressure were measured by standard methods described previously^16^. Body mass index (BMI) was calculated as weight (kg) divided by height in (m^2^). The economic status of each site was assessed by the local gross domestic product per capita in 2013. The 22 study sites were divided into high and low economic status per the national gross domestic product per capita in 2013^9^.

Venous blood samples were drawn after an overnight-fast of at least 8 h. After immediate centrifugation, the blood, serum, and plasma were frozen in a central laboratory certified by the College of American Pathologists. HbA1c was measured by high-performance liquid chromatography (MQ-2000PT, Medconn, China). Fasting plasma glucose and creatinine was measured by a Beckman Coulter AU 680 analyzer (USA).

### Exposure age

Exposure to famine was based on the year of birth. As stated in previous studies^9, 10, 12^, subjects were categorized into 4 groups according to their life stages when exposed to famine from 1st January 1959 to 31st December 1962: those born between 1959 and 1962 as fetal-exposed (*n* = 647); those born between 1949 and 1958 as childhood-exposed (*n* = 1679); those born between 1921 and 1948 as adolescence/young adult-exposed (*n* = 1003), and participants born between 1963 and 1974 as non-exposed (40–51 years) (*n* = 1795). The non-exposed (1963–1974) group was the reference.

### Definition of variables

The estimated glomerular filtration rate (eGFR) was calculated according to the Chronic Kidney Disease Epidemiology Collaboration (CKD-EPI) equation: for a woman with Scr ≤ 0.7 mg/dl, eGFR = 144 × (Scr/0.7)^−0.329^ × (0.993)^age^; for a woman with Scr > 0.7 mg/dl, eGFR = 144 × (Scr/0.7)^−1.209^ × (0.993)^age^. The value of eGFR is reported in units of mL/min per 1.73 m^2^ of body surface area^17^. CKD was defined as eGFR less than 60 mL/min per 1.73 m^2^^18^.

Diabetes was determined by history of diabetes, an fasting plasma glucose ≥7.0 mmol/L, or an HbA1c ≥ 6.5%.

### Statistical analysis

IBM SPSS Statistics, version 22 (IBM Corporation, Armonk, NY, USA) was used. All analyses were two-sided. A *P* value < 0.05 indicated a significant difference. Continuous variables were expressed as the mean ± SD, and categorical variables were described as a percentage (%). The variables were compared by analysis of covariance. Age adjusted *P* value was shown.

This is a sub-sample from a large ongoing cross-sectional study. When we set type 1 error rate at 5% and power at 0.80, this sample size could discover a 1.5 mL/min per 1.73 m^2^ difference of eGFR between the fetal-exposed and non-exposed, which was much greater than the difference in Table 1.

**Table 1:** Characteristics of study population by age of exposure to famine

	Fetal-exposed (1959–1962)	Childhood-exposed (1949–1958)	Adolescence/adult-exposed (1921–1948)	Non-exposed (1963–1974)
Age in 2014	52 ~ 55	56 ~ 65	66 ~ 93	40 ~ 51
*N*	647	1679	1003	1795
eGFR, mL/min per 1.73 m^2^	87.8 ± 13.0*	83.4 ± 12.3	74.9 ± 13.4	95.0 ± 12.3
Creatinine (μmol/L)	68.7 ± 11.8	68.7 ± 12.6	71.5 ± 17.5	66.6 ± 9.7
BMI (kg/m^2^)	24.9 ± 3.4*	25.1 ± 3.6	25.0 ± 3.8	24.0 ± 3.3
SBP (mmHg)	133.5 ± 19.9*	137.7 ± 21.9	145.7 ± 21.3*	125.2 ± 19.6
DBP (mmHg)	81.1 ± 12.8*	80.1 ± 12.7	79.8 ± 12.7*	77.0 ± 13.1
Diabetes (%)	11.7	18.2*	24.7	6.0
Rural/urban (%)	62.4/37.6^#^	62.2/37.8^#^	65.1/34.9^#^	56.6/43.4
Low/high ES (%)	37.9/62.1^#^	30.7/69.3^#^	34.0/66.0^#^	49.7/50.3

Continuous variables were expressed as the mean ± SD, and categorical variables were described as a percentage (%). The variables were compared by analysis of covariance

*eGFR* estimated glomerular filtration rate, *BMI* body mass index

^*^age-adjusted *P* < 0.05, significantly different from non-exposed (1963–1974)

^#^*P* < 0.05, significantly different from non-exposed (1963–1974)

We used linear regression analyses to measure the association between eGFR with life stages when exposed to famine. Model 1 was adjusted for age. Model 2 was adjusted for age, rural/urban residence, and economic status of areas. Model 3 was further adjusted for comorbidities or mediators including BMI, diabetes, and hypertension. Data were expressed as unstandardized coefficients (95% confidence interval). We further analyzed the relationship between life stages of famine exposure and risk of CKD in adulthood, binary logistic regression analysis was used. The models were the same as those in the linear regressions.

In sensitivity analyses, an equation for “Asian origin” was subsequently developed by the same group of investigators who developed the previous equation^19^. For a woman with Scr ≤ 0.7 mg/dl, eGFR = 151 × (Scr/0.7)^−0.328^ × (0.993)^age^; for a woman with Scr > 0.7 mg/dl, eGFR = 151 × (Scr/0.7)^−1.210^ × (0.993)^age^. We used this equation to reperform the logistic regression.

## Results

### Characteristics of participants

Table 1 summarizes the comparisons of the variables between the non-exposed and other life stages when exposed to famine. Compared with the non-exposed participants, the fetal-exposed had significantly lower eGFR (87.8 ± 13.0 vs. 95.0 ± 12.3 mL/min per 1.73 m^2^, age-adjusted *P* < 0.05), childhood-exposed and adolescence/adult-exposed had comparable eGFR after adjusting age (age-adjusted *P* > 0.05). The fetal-exposed also had significantly greater BMI (24.9 ± 3.4 vs. 24.0 ± 3.3 kg/m^2^, age-adjusted *P* < 0.05) and systolic blood pressure (133.5 ± 19.9 vs. 125.2 ± 19.6 mmHg, age-adjusted *P* < 0.05).

### Prevalence of CKD by life stages when exposed to famine

**Fig. 2:**
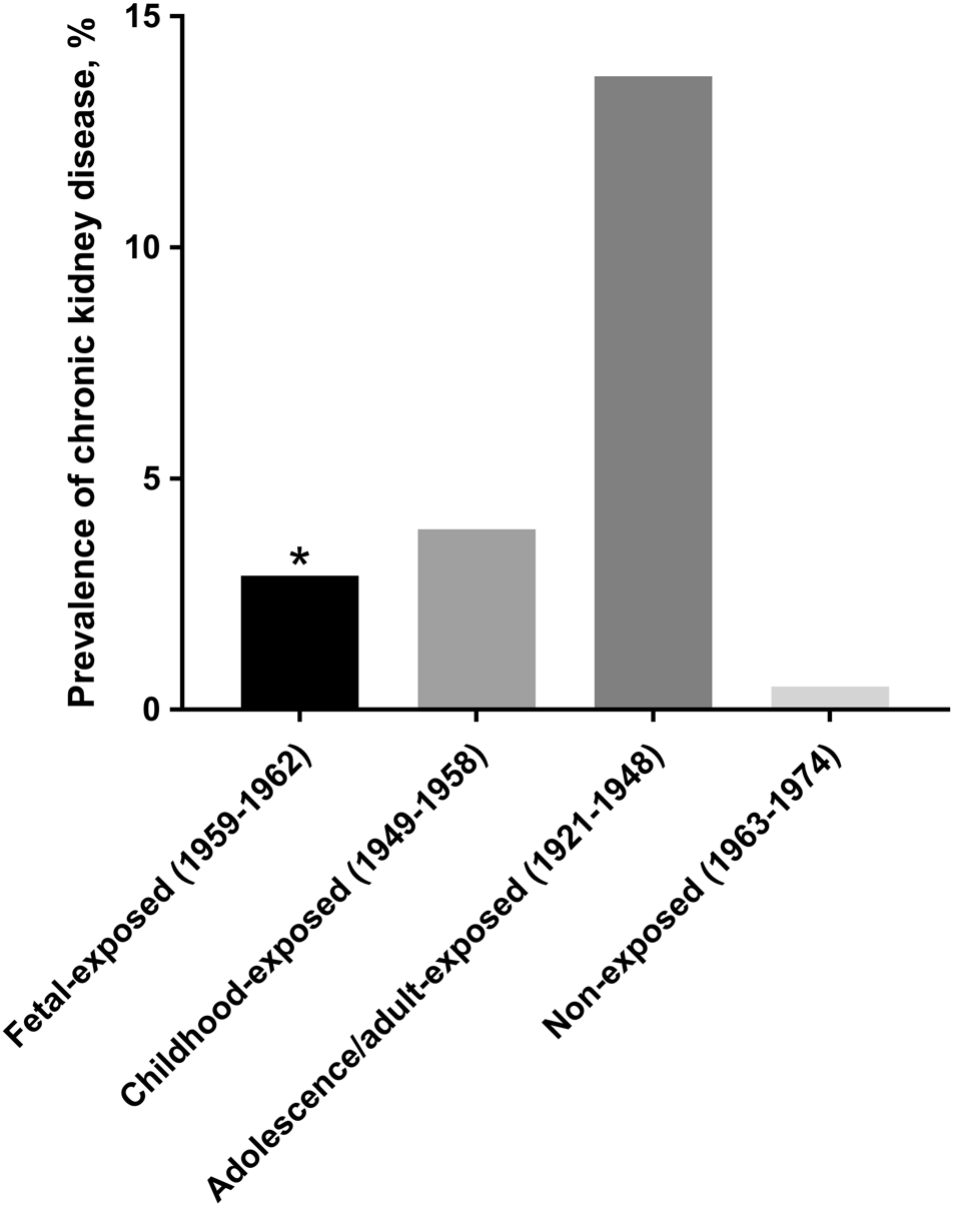
Prevalence of chronic kidney disease in different life stages when exposure to famine. *age-adjusted *P* value, compared with non-exposed (1963–1974)

The prevalence of CKD (eGFR < 60 mL/min per 1.73 m^2^) in the non-exposed, fetal-exposed, childhood-exposed, and adolescence/adult-exposed groups was 0.5, 2.9, 3.9, and 13.7%, respectively, (Fig. 2). In comparison with the non-exposed group, fetal-exposed women had a significantly higher prevalence of CKD (age-adjusted *P* < 0.05). The childhood-exposed and adolescence/adult-exposed did not show significant difference with the non-exposed after adjusting age (age-adjusted *P* > 0.05).

### Association of famine exposure with eGFR

Table 2 shows the results of the association between famine exposure and eGFR. Using the non-exposed group as the reference in fetal-exposed women there was a significant negative association with eGFR in crude model adjusting for age (B −1.47, 95% CI −2.81, −1.13, *P* < 0.05). In model 2, further adjustment for rural/urban residence and economic status did not attenuate the association (B −1.56, 95%CI −2.86, −0.25, *P* < 0.05). The association was attenuated in the final model further adjusting for BMI, diabetes, and hypertension, but remained significant (B-1.35, 95% CI −2.67, −0.04, *P* < 0.05).

**Table 2:** Association of famine exposure with eGFR and chronic kidney diseases

	Fetal-exposed (1959–1962)	Childhood-exposed (1949–1958)	Adolescence/adult-exposed (1921–1948)	Non-exposed (1963–1974)
eGFR				
Model 1	−1.47(−2.81, −1.13)^*^	−0.34(−2.03, 1.34)	−0.62(−3.34, 2.10)	Ref.
Model 2	−1.56(−2.86, −0.25)^*^	−0.06(−1.71, 1.59)	0.30(−2.35, 2.96)	Ref.
Model 3	−1.35(−2.67, −0.04)^*^	0.29(−1.38, 1.96)	0.89(−1.80, 3.59)	Ref.
CKD				
Model 1	2.85(1.25, 6.50)^*^	1.69(0.73, 3.88)	1.97(0.68, 5.75)	Ref.
Model 2	2.78(1.22, 6.36)^*^	1.52(0.66, 3.54)	1.58(0.53, 4.68)	Ref.
Model 3	2.42(1.05, 5.58)^*^	1.23(0.52, 2.90)	1.18(0.39, 3.59)	Ref.

Data are expressed as unstandardized coefficients (95%CI) in eGFR and odds ratios (95%CI) in chronic kidney disease. Linear regression analyses were used. eGFR, estimated glomerular filtration rate; CKD, chronic kidney diseases. CKD was defined as eGFR less than 60 mL/min per 1.73 m^2^

For a woman with Scr ≤ 0.7 mg/dl, eGFR = 144 × (Scr/0.7)−0.329 × (0.993)age; for a woman with Scr > 0.7 mg/dl, eGFR = 144 × (Scr/0.7)^−1.209^ × (0^.^993)^age^

Model 1 included age; Model 2 included age, rural/urban residence, economic status of areas; Model 3 included terms for model 2, BMI, diabetes and systolic blood pressure

^*^ indicated *P* < 0.05

Using an equation for Asian origin, the results were rather similar. In the final model, the association was significant in the fetal-exposed (B −1.42, 95% CI −2.80, −0.04, *P* < 0.05) (Table 3, model 3).

**Table 3:** Association of famine exposure with eGFR and chronic kidney diseases using an equation especially for Asian

	Fetal-exposed (1959–1962)	Childhood-exposed (1949–1958)	Adolescence/adult-exposed (1921–1948)	Non-exposed (1963–1974)
eGFR				
Model 1	−1.39(−2.81, 0.03)	−0.05(−1.84, 1.75)	−0.13(−3.03, 2.78)	Ref.
Model 2	−1.45(−2.83, −0.07)^*^	0.32(−1.43, 2.07)	1.01(−1.82, 3.84)	Ref.
Model 3	−1.42(−2.80, −0.04)^*^	0.31(−1.45, 2.06)	0.94(−1.89, 3.76)	Ref.
CKD				
Model 1	3.99(1.25, 12.74)^*^	2.43(0.77, 7.73)	2.90(0.72, 11.78)	Ref.
Model 2	3.91(1.22, 12.49)^*^	2.20(0.69, 7.07)	2.29(0.56, 9.47)	Ref.
Model 3	3.73(1.16, 11.94)^*^	2.03(0.63, 6.55)	2.06(0.50, 8.59)	Ref.

Data are expressed as unstandardized coefficients (95%CI) in eGFR and odds ratios (95%CI) in chronic kidney disease. Linear regression analyses were used. eGFR, estimated glomerular filtration rate; CKD, chronic kidney diseases. CKD was defined as eGFR less than 60 mL/min per 1.73 m^2^

For a woman with Scr ≤ 0.7 mg/dl, eGFR = 151 × (Scr/0.7)−0.328 × (0.993)age; for a woman with Scr > 0.7 mg/dl, eGFR = 151 × (Scr/0.7)^−1.210^ × (0^.^993)^age^

Model 1 included age; Model 2 included age, rural/urban residence, economic status of areas; Model 3 included terms for model 2, BMI, diabetes and systolic blood pressure

* indicated *P* < 0.05.

### Association of famine exposure with CKD

Table 2 also summarizes the association between famine exposure and CKD (eGFR < 60 mL/min per 1.73 m^2^). In accord with the association between eGFR and famine exposure, fetal exposure was significantly associated with CKD after adjusting for age (OR 2.85, 95% CI 1.25, 6.50, *P* < 0.05) (Table 2, model 1). Further adjusting for rural/urban residence, economic status of areas, BMI, diabetes, and hypertension did not change the association significance (OR 2.32, 95% CI 1.01, 5.36, *P* < 0.05) (Table 2, model 3). In the sensitivity analysis, fetal exposure was still significantly associated with CKD (OR 3.73, 95% CI 1.16, 11.94, *P* < 0.05) (Table 3, model 3).

## Discussion

In this study, we found that individuals who were exposed to famine in fetal period had lower eGFR and higher prevalence of CKD (eGFR < 60 mL/min per 1.73 m^2^) in adulthood, compared with those who had not been exposed to famine. Exposure to famine during childhood and adolescence did not present this association. Adjusting for variables including age, adiposity, diabetes, and hypertension did not alter this association. These results indicated that fetal malnutrition was linked to impaired kidney function in the adulthood.

In previous studies, we found people, especially women who were exposed to famine in early life had greater risk of diabetes^9^, non-alcoholic fatty liver disease^11^, and metabolic syndrome^12^. Currently, we have results that show exposure to famine in fetal period is associated with declined kidney function and CKD. This distinct relation provides further evidence that an adverse nutrition event has long-lasting impact on the risk for chronic disease development later in life^6^. We should bear in mind that malnutrition is early life is still very common globally. Maternal undernutrition (BMI less than 18.5 kg/m^2^) ranges from 10 to 19% in most countries^20^. Twenty percent of children younger than 5 years in low-income and middle-income countries had underweight, mostly because of nutrition^20^. Thus, malnutrition in early life and its consequences are still important topics to be further explored and tackled.

Our findings were consistent with previous studies on malnutrition in early life and kidney function. The pioneer study base on Dutch famine found people who were exposed to famine in mid gestation had 2 times higher incidence of microalbuminuria (defined as albumin/creatinine ratio > 2.5) (OR 2.1, 95% CI 1.0, 4.3) and after adjusting confounders such as adult weight, blood pressure, fasting blood glucose, economic status, and size at birth did not change this association (OR 3.2, 95% CI 1.4, 7.7)^13^. Another intriguing study from China’s Great Famine also indicated famine exposure was linked to a greater risk (OR 1.54, 95% CI 1.04, 2.28) of higher proteinuria level measured with a dipstick test of random urine specimens among women born during the famine years (1959–1961)^6^. Other common surrogate markers for intrauterine conditions were LBW, small for gestational age and premature birth. Newborns, children and young adult having suffered from the above conditions often have reduced GFR^21–23^. Particularly, LBW was associated with 73, 58, 81, and 79% higher risk of CKD, end-stage renal disease, albuminuria and reduced GFR, respectively^14^. Even in young adult, LBW was also associated with enhanced risk of CKD (OR 2.88, 95% CI 2.28, 3.63) and reduced GFR (OR 6.36, 95% CI 4.00, 10.12)^24^. These epidemiological studies indicate an intrinsic and direct relationship may exist between prenatal famine exposure and renal dysfunction in later life.

Then what is the possible underlying mechanism in this intrinsic relationship? The impaired nephrogenesis that resulted from the famine exposure may be the predominant reason^6, 25^. Under normal developmental conditions, nephrogenesis starts from about 4th to 5th week of gestation and continues until the 36th week of gestation^26^. Prenatal undernutrition results in developmental disruption in Notch signaling pathway, an important pathway in nephron formation^27^. Maybe that’s why the increased risk of CKD and declined GFR existed in fetal famine exposure but not in childhood or young adulthood in our study. More accurately, the Dutch famine study found only people who were exposed to famine in mid gestation had higher rates of microalbuminuria^13^. That may be because of sharp increase of nephrons from the 18th to 32nd week^28^. Animal studies (nephron numbers can be counted only postmortem in human) showed less number of nephrons led to renal dysfunction in later life^29, 30^. In human being, from data of postmortem, Hughson, M. et al. found there was an decrease of about 250,000 glomeruli when the birth weight decreases each kilogram^31^. Furthermore, reduced kidney mass, which was proportional to nephron number^32, 33^, was associated with LBW in different age and population groups^25, 34^. Lighter kidney mass, less nephron number indicated bigger glomerular size, thus increasing the risk of glomerular hyperfiltration, glomerulosclerosis and further CKD^25, 35^.

Our study has some strengths. First, this is the first study to measure the effects of the China’s Great Famine 1959–1962 on GFR and CKD in adults who were prenatally exposed to famine about six decades ago. Second, SPECT-China study had strong quality control. All the investigation in each site was performed by the same trained group. All the biochemical measurements were done in one central laboratory certified by the College of American Pathologists. Finally, we recruited general population in various areas in East China, so the results may be more representative than other clinic-base studies.

This study also has some limitations. First, as we mentioned in previous studies^10^,we assumed that the subjects were born in the local province or area and did not move out. Actually, permanent residency acquisition still has strict requirements and only a small proportion of rural population lived in provinces other than their birthplaces^36^. Furthermore, the prenatally exposed subjects are 52–55 years., which is close to retirement age, so they are less likely to be mobile. Second, GFR was estimated by CKD-EPI equation, not by inulin urinary clearance. However, this approach is too costly and may not feasible in large epidemiologic studies^37^. Third, creatinine was obtained based on single measurement, so the prevalence of CKD might be overestimated^3^.

In conclusion, prenatal exposure to the Great Famine was associated with declined eGFR and greater risk of CKD (eGFR < 60 mL/min per 1.73 m^2^) in women, which indicated that famine in early life may play a role in the development of kidney dysfunction. Combining with our previous studies^9–12^, this suggests that prenatal and early postnatal-time window require the highest priority in obtaining nutritional relief.
